# Genetic Diversity and Levels of Expression of Factor H Binding Protein among Carriage Isolates of *Neisseria meningitidis*

**DOI:** 10.1371/journal.pone.0107240

**Published:** 2014-09-23

**Authors:** Ludovic Lemée, Eva Hong, Manuel Etienne, Ala-Eddine Deghmane, Valérie Delbos, Aude Terrade, Gilles Berthelot, Francois Caron, Muhamed-Kheir Taha

**Affiliations:** 1 Centre Hospitalier Universitaire, Rouen, France; 2 Institut Pasteur, National de Reference Centre for meningococci and Invasive Bacterial infections Unit, Paris, France; 3 Centre Hospitalier Général, Dieppe, France; University of Kentucky College of Medicine, United States of America

## Abstract

The prevention of meningococcal disease may be improved by recombinant vaccines such as 4CMenB and rLP2086 that target the factor H binding protein (fHbp), an immunogenic surface component of *Neisseria meningitidis* present as one of three variants. Whether such vaccines decrease carriage of invasive isolates and thus induce herd immunity is unknown. We analyzed the genetic diversity and levels of expression of fHbp among 268 carriage strains and compare them to those of 467 invasive strains. *fhbp* gene sequencing showed higher proportions of variants 2 and 3 among carriage isolates (p<0.0001). Carriage isolates expressed lower levels of fHbp (p<0.01) but that remain high enough to predict targeting by antibodies against fHbp particularly in group B isolates belonging to the frequent hypervirulent clonal complexes in Europe and North America (cc32, cc41/44, cc269). This suggests that fHbp targeting meningococcal vaccines might reduce, at least in part, the acquisition of some hyperinvasive isolates.

## Introduction

Invasive meningococcal diseases (IMD) such as meningitis and meningococcemia remain in all countries one of the leading cause of death from bacterial infection in otherwise healthy young subjects [1]. While meningococcal carriage in the nasopharynx is common (estimated to concern about 10% of the general population [2]), invasion is a rare phenomenon, excepted during outbreaks. Numerous studies have shown that disease isolates (i.e. isolates from IMD cases) differ from carriage isolates, belonging to a limited number of genetic lineages or clonal complexes (cc) known for their virulence [3]. For example, in Europe and North America, five clonal complexes (cc8, cc11, cc32, cc41/44 and cc269) are responsible for most IMD cases [3], [4]. Usually, IMD occurs upon a recent acquisition of such pathogenic isolates that escape innate host immunity (complement mediated bacterial lysis and phagocytosis) due to meningococcal virulence factors such as the capsule and the ability to bind negative regulators of complement pathway [5].

Most IMD in Europe, America and Oceania are due to group B isolates [6]. Their prevention might be revolutionized at short term by a recently licensed recombinant vaccine containing 4 components (4CMenB/Bexsero) that is expected to offer a broad group B isolates coverage [7]. Another bivalent recombinant vaccine (rLP2086) is also under development [8]. Both of these vaccines target the factor H binding protein (fHbp), an immunogenic surface component known to contribute to the virulence of *Neisseria meningitidis* through binding of the negative regulator of the alternative pathway of the complement [5]. Because fHbp also exists in non group B meningococcal isolates, a coverage of some of these isolates is expected, as recently shown for 4CMenB/Bexsero against some group X isolates (from Africa but not from Europe) [9].

One key-question for these fHbp-based vaccines concerns their ability to decrease meningococcal acquisition/carriage of invasive isolates and thus to induce herd immunity, as previously shown for conjugate meningococcal C vaccine.

Carriage studies currently performed among subjects receiving 4CMenB/Bexsero or rLP2086 (clinicalTrials.gov identifier NCT01214850) will probably help answering the question, providing they will not be biased by a local ecology that do not necessary reflect the global epidemiology. An alternative approach to anticipate the potential herd immunity effect of recombinant meningococcal vaccines can be achieved by analyzing the genetic diversity and levels of expression of fHbp among current carriage isolates. Indeed, the protective effect against carriage of meningococcal recombinant vaccines is supposed to be dependent on the cross-reactivity with the vaccine fHbp and the level of expression of fHbp in virulent isolates in sitting of carriage, the higher the expression, the higher the protection is.

Thus, the aim of this study was to analyze the fHbp expression among carriage meningococcal isolates currently encountered in France, and to compare them to those observed among disease isolates.

## Methods

### Bacterial isolates and ethical statement

#### Collection of carriage isolates

Carriage isolates came from a study conducted in the early 2008 during a clonal B:14:P1.7,16/ST-32 meningococcal outbreak that had affected a part of the French Normandy region from 2003 to 2010 [10]. This carriage study has been previously detailed [11]. It was approved by the Regional Ethical Committee for Medical Research (Comité de Protection des Personnes Nord-Ouest, CPP NordOuest) as well as the National French Data Protection Agency (Commission Nationale Informatique et Liberté). Written informed consent was obtained from parents or legal guardian of each participant. Out of 3522 volunteers aged 1–25 years, 196 had a detectable pharyngeal carriage of *N. meningitidis* (all isolates included), with only one isolate detected by subject (i.e., no detection of polyclonal carriage). Among these 196 subjects, 112 had a second swab, in a mean delay of 6.7 days after the first (range, 4–12 days); most of them (87/112, 78%) still carried a *N. meningitidis* isolate, which was identical or closely related to the isolate of the first swab for almost all subjects (see details below).

Because some isolates were not recovered after subculture, the final collection of carriage isolates included a total of 268 isolates that corresponded to 188 subjects. For these subjects, 101 had only primary-swab isolates, 80 had both primary and secondary swabs isolates, and 7 subjects had only secondary swab isolates. Given the extremely low level of carriage of the epidemic B:14:P1.7,16/ST-32 strain (see below), this collection was supposed to well reflect the isolates responsible for healthy meningococcal carriage at the country level [11].

#### Collection of disease isolates

Disease isolates came from the collection of the National Reference Centre for Meningococci (NRCM) at the Pasteur Institute. For that collection the NRCM had approvals from the internal board of the Institut Pasteur to collect, characterize and use these samples that are all anonymized, and that of the National French Data Protection Agency (Commission Nationale Informatique et Liberté). To avoid the overrepresentation of B:14:P1.7,16/ST-32 observed in Normandy (which represented up to 80% of the total disease isolates at the acme of the outbreak) all the 487 culture-confirmed disease isolates collected in France in 2008 were analyzed. Such collection reflects well the isolates responsible for IMD at the country level. Indeed, in France, for each case of IMD, it is requested that the isolate at a local level is send to the NRCM for complete typing.

### Phenotypic and genotypic characterization of meningococcal isolates

All the carriage and disease isolates were typed at the NRCM in cooperation with the Rouen University hospital. They were grown under 5% CO_2_ on GCB agar plate with Kellogg supplements [12], and characterized by phenotyping (serogroup:serotype:serosubtype), antibiotic susceptibility testing and genotyping by both capsule gene amplification, multilocus sequence typing (MLST), pulse-filed gel electrophoresis (with two restriction enzymes, Nhe1 and SpeI) and *porA* typing [13], [14], [15]. Antibiotic susceptibility testing to define the minimal inhibitory concentrations for penicillin G, cefotaxime, ceftriaxone, ciprofloxacin and rifampicin was performed using Etest (BioMérieux, Marcy l'Etoile, France) on Mueller-Hinton agar plates supplemented with sheep blood and interpreted as previously described [15]. Full MLST data (sequence types and clonal complexes) were obtained for 126 disease isolates (each fourth isolate representing 25% of the total 487 isolates). For the carriage isolates full MLST data were obtained for 166 isolates.

### fHbp analysis

For the total set of carriage isolates (268 isolates), the genetic diversity and levels of expression of meningococcal factor H binding protein were analyzed, using methods previously described [16]. The sequence of *fHbp* gene was performed to determine the distribution of the corresponding allele using primers and sequencing methods that we have previously reported [16].

The level of the expression of fHbp was quantified by ELISA and using anti-fHbp antibodies directed against both subfamilies (fHbp variant 1/family B and variant 2 or 3/family A). These antibodies were obtained in rabbits that were immunized with purified fHbp. After immunization, antibodies were purified from rabbit sera, and then used in ELISA tests as previously described [16]. The results obtained with the carriage isolates were compared to those previously observed for the disease isolates which have been published elsewhere [16].

Susceptibility to anti-fHbp antibodies of the selected strains was also analysed. Titration of bactericidal activity was performed using the previously described anti-fHbp IgG that are directed against variants 1, 2 and 3 of fHbp [16]. Assays were performed with several carriage isolates and a human serum as an exogenous complement source as previously described [10]. The titers corresponded to the reciprocal of the final serum dilution causing 50% killing of the inoculum. Bactericidal titers lower than 4 were assigned a value of 2.

### Data analysis

Isolates for which the expression of an A, B, C, Y, W, X, or E capsule was detected by agglutination were designated as “serogroupeable isolates”. Isolates were also tested by PCR for the specific genes serogroups A, B, C, Y, W, or X; isolates for which the PCR was positive were designated as “genogroupeable isolates”.

The identification of sequence type (ST) and clonal complexes was performed through (http://pubmlst.org/neisseria/). Relationships and clustering of typing data were performed using the START package available through (http://pubmlst.org) [17]. Alleles of *fHpb* were described according to their classification into two subfamilies or three variants [18]. The expression of fHbp was analysed using the optic densities (OD) obtained by ELISA after substraction of the background OD (a negative isolate for fHbp). The results were expressed as a ratio over that of the positive control and were analyzed using Z Score transformation as previously described [16]. OD data were log transformed and normalized to the mean of log ratios of all ODs measures and then expressed as unit of standard deviation (SD) above/below the mean of log ratios, a Z score ≤−2 corresponding to low expression of fHbp.

For the comparison between carriage isolates and diseases isolates, only one isolate by patient was retained (i.e., the doubloons of the carriage isolates set were excluded to avoid an overrepresentation at the corresponding isolates).

Data were analyzed using the Chi-squared test, Student's *t*-test and analysis of variance (ANOVA). A *P* value of ≤0.05 was considered to be statistically significant. When Chi-squared test involved several comparisons, the Bonferroni correction was applied.

## Results

### 1- Overall phenotypic and genotypic diversity of carriage isolates compared to disease isolates

Higher diversities of phenotypes and genotypes were observed for the carriage isolates, compared to disease isolates, as summarized in Table 1.

**Table 1:** Overall phenotypic and genotypic diversity of *N meningitis* isolates from France in 2008, either from healthy carriers (i.e., carriage isolates) or from cases of invasive meningococcal disease (i.e., invasive isolates).

	Carriage isolates	Disease isolates	p
Serogrouping:	n = 188	n = 487	
non serogroupeable isolates	92 (48.9%)	3 (0.6%)	<0.007*
serogroupeable isolates	96 ((51.1%)	484 (99.4%)	<0.007*
serogroup B	46 (24.5%)	318 (65.3%)	<0.007*
serogroup C	16 (8.5%)	115 (23.6%)	<0.007
serogroup Y	16 (8.5%)	30 (6.2%)	ns
serogroup W	6 (3.2%)	21 (4.3%)	ns
serogroup X	4 (2.1%)	0 (0%)	<0.007*
others	8 (4.3%)	0 (0%)	<0.007*
Genogrouping:	n = 188	n = 487	
non genogroupeable isolates	69 (36.7%)	0 (0%)	<0.008#
genogroupeable isolates	119 (63.3%)	487 (100%)	<0.008#
genogroup B	67 (35.6%)	319 (65.5%)	<0.008#
genogroup C	24 (12.8%)	116 (23,8%)	<0.008#
genogroup Y	16 (8.5%)	31 (6.3%)	ns
genogroup W	7 (3,7%)	21 (4.3%)	ns
genogroup X	5 (2.7%)	0	<0.008#
Serosubtyping:	n = 188	n = 487	
non serosubtypeable isolate	24 (12.8%)	99 (20.3%)	ns
serosubtypeable isolate	164 (87.2%)	388 (79.7%)	ns
number of serosubtypes	19	20	
Clonal complexes (cc):	166	126	
5 hyperinvasives European clonal cc	56 (33.7%)	98 (77.8%)	<0.008#
cc8	0	0	
cc 11	13 (7.8%)	35 (27.8%)	<0,008#
cc 32	19 (11.4%)	13 (10.3%)	ns
cc 41/44	21 (12.7%)	40 (31.7%)	<0.008#
cc 269	3 (1.8%)	10 (7.9%)	ns
Genetic diversity. Simpson's Index	0.974	0.901	0.002
95% Confidence Interval	0.963–0.942	0.859–0.942	
Antibiotic susceptibility testing; susceptibility to:	n = 188	n = 487	
penicillin G	124 (66%)	380 (78%)	0.007
cefotaxime/ceftriaxone	188	487	
rifampicin	188	487	
ciprofloxacin	188	487	

*Bonferroni correction for 7 comparisons.

#Bonferroni correction for 6 comparisons.

ns: non significant.

**Figure 1:**
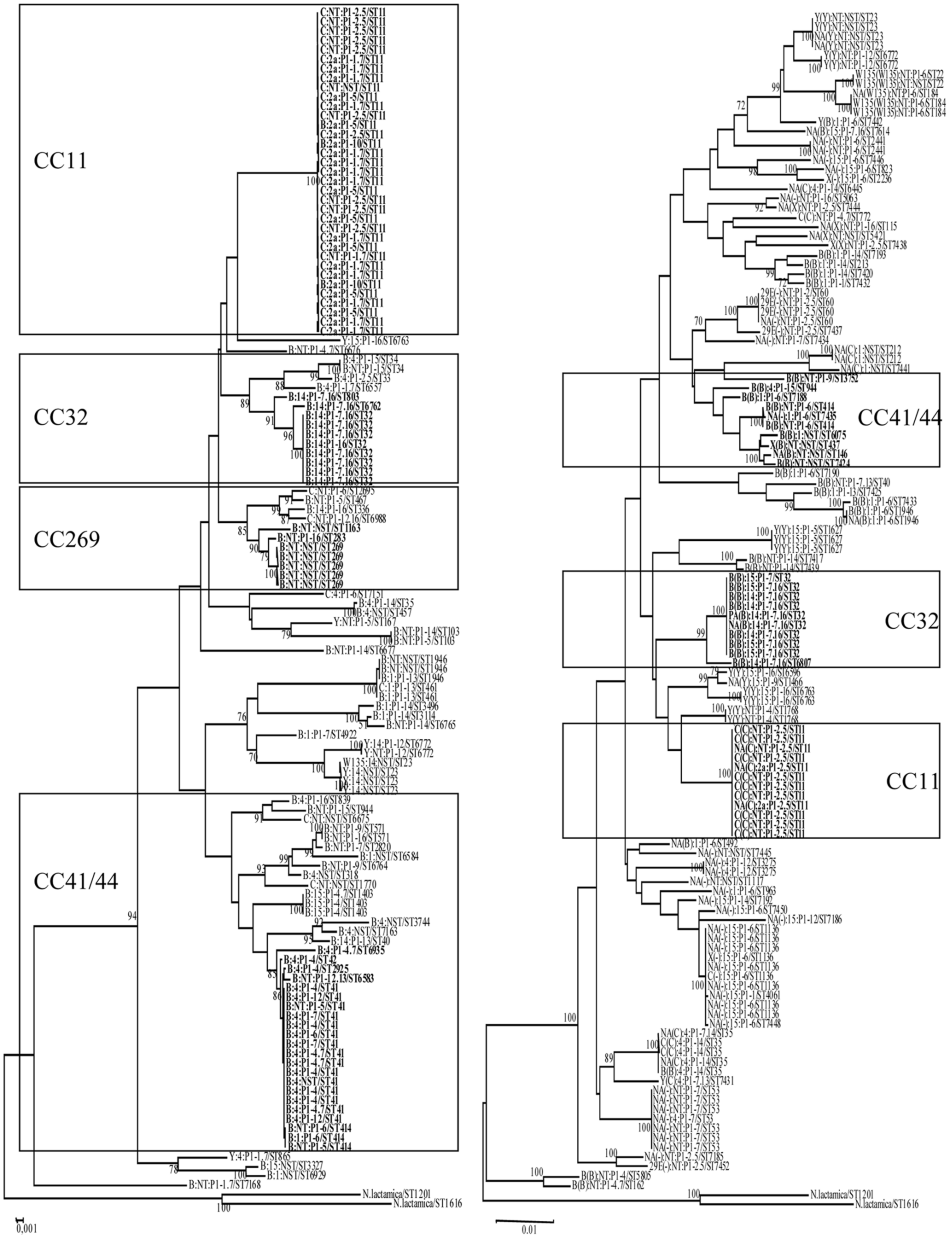
Phylogenetic relationships between carriage and invasive isolates. On the right side of the figure the carriage isolates are displayed (n = 124). The invasive isolates for which full MLST data were available (n = 126) are on the left side of the figure. Relationships and clustering of typing data were performed using the START package available through (http://pubmlst.org).

While all invasive isolates were capsulated, i.e., were serogroupeable, only half of the carriage isolates (96/188, 51.1%) showed a serogroupable phenotype. However, among the 92 non serogroupeable carriage isolates, 35 (18.6% of the total set) were genogroupeable, suggesting that such isolates could be potentially virulent, should they be able to recover the expression of capsule genes. Among the remainder 57 non genogroupeable carriage isolates, 41 (21.8% of the total set) were positive for null capsule locus (*cnl*), suggesting a total absence of virulence. The carriage isolates also showed significantly higher diversity in terms of combinations serotypes/serosubtypes (Table 1). Among the 166 carriage isolates for which a sequence type was determined, 84 sequence types were identified that belonged to 22 different clonal complexes. Only 33.7% of these isolates belonged to one of the five hyperinvasive clonal complexes commonly encountered in Europe (cc8, cc11, cc32, cc41/44 and cc 269). At the opposite, 77.8% of the disease isolates belonged to these 5 hyperinvasive clonal complexes (p<0.008). As a result the overall genetic diversity was significantly higher for carriage isolates than for disease isolates (Simpson's index of diversity 0.974 and 0.901, respectively, p = 0.002), as shown in Fig. 1.

All the carriage and disease isolates were susceptible to ciprofloxacin, rifampicin and cefotaxim. The proportion of isolates with reduced susceptibility to penicillin G (MIC ≥0.125 mg/L) was significantly higher for the carriage isolates than for the disease isolates (34% versus 22%, p = 0.007).

### 2- fHbp analysis

Data of *fHbp* gene sequencing were obtained for 187 out of the 188 carriage isolates; they showed 28 different DNA alleles of fHbp that encodes 20 different peptides. All fHbp alleles are indicated in the Table S1 and full sequences are available on PubMLST database (http://pubmlst.org/neisseria/) through the dropmenu (fHbp_PEPTIDEfrag_Pasteur and fHbp_DNAfrag_Pasteur). Based on protein sequence data, 51 (27.3%) isolates corresponded to variant 1 of fHbp, 93 (49.7%) corresponded to variant 2 of fHbp and 43 (23%) belonged to variant 3. The proportion of variant 1 fHbp among invasive isolates (regardless the serogroup) was 58%, a proportion significantly higher (p<0.0001) from that observed among carriage isolates. The most frequent *fHbp* allele among carriage isolates was *fHbp11* that encoded peptide9/variant 2. The peptide fHbp9 accounted for 15.5% (n = 29) of peptides of the carriage isolates, while it was of lower frequency among invasive isolates (5.9%). This allele encodes a fHbp harbouring the residues that are involved in binding to human factor H [19].

The expression levels of fHbp at the bacterial surface were analyzed in all the 188 carriage isolates. Fifty seven isolates (30.3%) did not show any detectable fHbp. The remaining 131 isolates were classified according to their distribution around the mean value of fHbp ratios (detailed scores are displayed in the Table S1). Three isolates showed Z scores that were ≤−2 standard deviations below the mean. Thus 60 (32%) carriage isolates were considered as fHbp negative isolates (57 with no detection and 3 with low score). The group distributions (serogroups and genogroups) of the carriage isolates according to status of expression of fHbp is showed in the Table 2. Group C isolates were significantly more present among fHbp-negative isolates, and this seems impacted by the role of the clonal complex ST-11; indeed, among the 13 group C carriage isolates belonging to the clonal complex ST-11, only two were fHbp positive (Z score >−2).

**Table 2:** Distribution of groups and clonal complexes (numbers and percentages) of carriage isolates according to their levels of production of fHbp.

	fHbp-negative isolates (n = 60, 32%)		fHbp-producing isolates n = 128, 68%)		
	Serogroup	Genogroup	Serogroup	Genogroup	p
B	15 (25%)	19 (31.7%)	31 (24.2%)	47 (36.7%)	ns
C	13 (21.7%)	14 (23.3%)	3 (2.3%)	10 (7.8%)	≤0.01#
Y	11 (18.3%)	12 (20%)	5 (3.9%)	4 (3.1%)	≤0.01#
W	0 (0%)	0 (0%)	6 (4.7%)	7 (5.5%)	ns
NG and others	21 (35%)	15 (25%)	83 (64.9%)	60 (46.9%)	≤0.01#
All hyperinvasive cc	16 (26.7%)		40 (31.2%)		ns
cc32, cc41/44, cc269	5 (8.3%)		38 (29.7%)		0.0125*
cc11	11 (18.3%)		2 (1.7%)		0.0125*
other cc	44 (73.3%)		88 (68.8%)		ns

# Bonferroni correction for 5 comparisons.

*Bonferroni correction for 4 comparisons.

ns: non significant.

**Figure 2:**
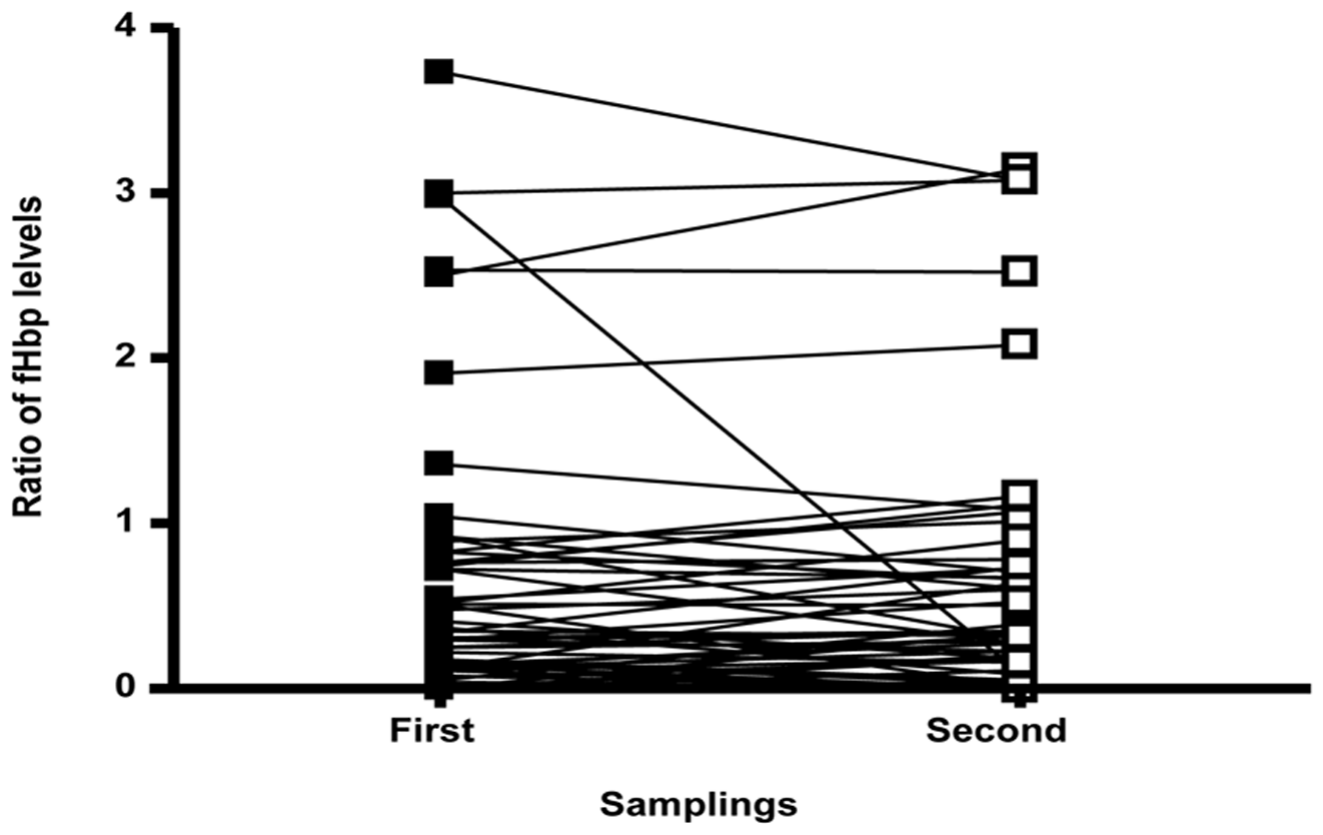
Schematic representation of the levels of expression of fHbp overtime among carriage isolates. Data were obtained from 80 subjects for whom two positive pharyngeal swabs were available at a mean of 1 weak interval. For each subject, the data were expressed as ratio of the expression of fHbp from the fist sample and the reference isolate and linked by a solid line to the ratio of the expression of fHbp from the second sample and the reference isolate.

As shown in Table 3 and Figure 2, the global levels of fHbp expression were significantly lower in carriage isolates than in disease isolates. This was also true when the isolates were considered according to their clonal complexes. Indeed, carriage isolates belonging to clonal complex cc11 or cc41/44 showed significant lower levels of fHbp compared to the invasive isolates belonging to the respective same clonal complex. In contrast, the differences in terms of fHbp expression between carriage and disease isolates was not statistically significant for isolates of the clonal complex cc269 (with the limit of low numbers), for isolates belonging to the cc32 or for isolates that did not belong to any known clonal complexes.

**Table 3:** Values of ratios of fHbp expression level among carriage and invasive isolates.

	Mean fHbp expression ratio (IC 95%)		
Clonal complexes	Carriage isolates (n = 188)	Disease isolates (n = 102)	p
cc11	0.014 (−0.007–0,036)	0.73 (0.49–0.97)	0.0002
cc32	1.7 (1.1–2.2)	2.6 (1.8–3.5)	0.0182
cc41/44	0.39 (0.23–0.54)	0.97 (0.78–1.2)	0.0001
cc269	0.97 (−1.3–3.3)	1.3 (0.62–2)	0.7143
Others	0.3 (0.23–0.37)	0.37 (0.18–0.57)	0.9875
All	0.43 (0.34–0.53)	0.92 (0.74–1.1)	<0.0001

*p* value ≤0.008 for significance (Bonferroni correction for 6 comparisons).

Table 4 shows the susceptibility to rabbit anti-fHbp antibodies of 10 carriage isolates expressing fHbp at different levels. No killing was observed for the isolates with low or no fHbp. In contrast, a carriage isolate that expressed fHbp at high level was susceptible to the fHbp antibodies, with high bactericidal titers (i.e., 16).

**Table 4:** Characteristics of carriage isolates and their susceptibility to anti-fHbp rabbit IgG.

Isolate	Group	Clonal complex	fHbp peptide	fHbp DNA	fHbp variant	Z score	survival titre
2995	B	cc213	13	18	3	ND	2
2934	B	cc213	13	18	3	ND	2
2371	B	cc213	13	18	3	ND	2
2716	B	cc35	9	11	2	ND	2
1046	C	cc254	12	3	1	ND	2
1231	B	cc213	13	18	3	ND	2
2794	C	cc11	12	3	1	ND	2
3601	B	cc162	10	16	2	−4.42	2
341	B	cc41/44	1	24	2	−2.21	2
3180	B	cc32	3	31	1	1.87	16

ND: non detectable.

### 3- Variation of carriage at one week interval

**Figure 3:**
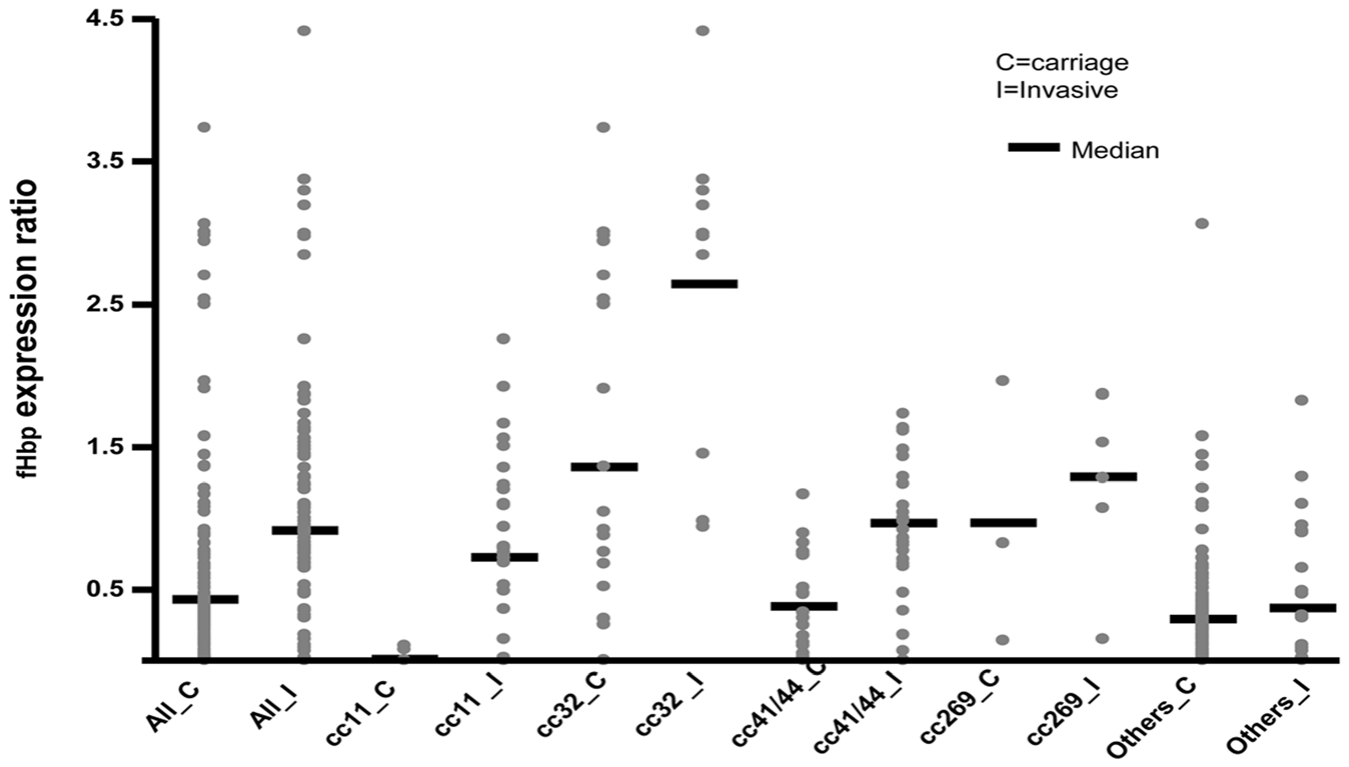
Distribution of relative levels of expression of fHbp among carriage and invasive isolates. Data were expressed as ratios of the expression for each strain compared to a reference strain a scatter plots (filled gray circles) and mean values (black lines). The clonal complexes (cc) to which belong the tested isolates are indicted under each group of scatter plots.

Among 80 subjects for which two positive pharyngeal swabs were available (at a mean of 6.7 days interval), 48 (60%) had a second isolate strictly identical to the first one, and 30 (37.5%) had a second isolate that differed but slightly by either their sequence type (but within the same cc), group, serotype or serosubtype. For only two subjects (2.5%), the second isolate was clearly different from the first one, both by its group and clonal complex. The expression levels of fHbp at the bacterial surface were available for the pairs of isolates of the 80 subjects. Overall, there was no significant difference (Fig. 3). A marked decrease in fHbp levels was observed for the second isolate in only two cases: a 4 time lower level for a pair of isolates that were genetically related (same cc162) and a 32 times lower level for one of the two subjects for which the second isolate was different.

Interestingly, one subject was carrier of the epidemic strain which was replaced at the second sampling by a non-groupeable isolate of the cc1136 (ST-7454) that also expressed a different fHbp allele.

## Discussion

We reported here the first study that had analyzed the levels of surface expression of fHbp among *N. meningitidis* isolates from healthy carriers, and compared them to those observed among invasive isolates collected in the same period on a same country. This fHbp expression is expected to be critical for the bacterial coverage of the new recombinant meningococcal vaccines, which are supposed to act not only by decreasing the risk of IMD at the individual level among immunized subjects, but also by avoiding the decreasing acquisition/transmission of virulent strains and thus to induce herd immunity [20].

In accordance with previous studies [2], [21], the carriage isolates here studied displayed higher heterogeneity than invasive isolates for almost all the investigated parameters including group, sequence type, and antibiotic susceptibility profile. In particular, there was a lower proportion of isolates belonging to the hyperinvasive clonal lineages, as shown by the comparison of the respective dendogram and diversity coefficient of each set (Fig. 1).

Carriage isolates were also very heterogenic for fHbp in terms of both DNA sequences and level of expression. While a majority (60%) of invasive isolates harbored fHbp variant 1, carriage isolates were most harmoniously distributed between the three variants; such results are in accordance with a previous work having suggest higher proportion of variants 2 and 3 among invasive *N. meningitides* strains [22]. Also, high sequence diversity of fHbp was observed among carriage isolates with 28 unique *fHbp* alleles corresponding to 20 unique fHbp proteins.

The most original data of that study concerned the levels of fHbp expression which were globally lower among carriage isolates than invasives isolates. For that analysis we used a tool previously described by the NRCM [16], i.e., rabbit anti fHbp antibodies that allowed ELISA quantification of fHbp at the surface of meningococcal isolates and to correlate these levels to the bactericidal effect of anti-fHbp antibodies. The proportion of isolates with no or low levels of fHbp did not differ significantly among carriage and invasive isolates (32% versus 24% p = 0.13). Such an absence of detectable fHbp seems to prevent the clearance of bacteria by anti-fHbp antibodies (Table 4). Of note, for isolates that expressed fHbp, the level of such expression was globally lower for carriage isolates, but remaining high enough for most hyperinvasive clonal complexes of serogroup B isolates (cc32, cc41/44 and cc269) to expect targeting by antibodies directed against fHbp [16]. This suggests that fHbp based vaccines such as 4CMenB/Bexsero or the bivalent rLP2086 vaccine might decrease nasopharyngeal acquisition and carriage of numerous invasive isolates, and thus induce herd immunity. In addition, fHbp was expressed among carriage isolates regardless the serogroup, confirming that « B recombinant vaccines » would be in fact « universal meningococcal vaccines » [23].

Our results clearly indicate that the levels of expression of fHbp could vary significantly. The importance of this observation may be related to the recent description of thermoregulation that has been suggested to increase the expression of fHbp by the meningococcus when temperature in the nasopharynx increased upon flu infection and hence triggers invasiveness [24]. Carriage isolates, at the opposite of invasive isolates, may not undergo this thermoregulation and retain lower fHbp levels.

In our opinion, evaluating the levels of vaccine-targeted antigens is crucial for studies that aim to address the impact of the new recombinant vaccines on carriage isolates. For the record, the impact of the meningococcal serogroup C conjugate vaccines was observed to be mainly on isolates belonging to the ST-11 clonal complex serogroup C and was suggested to be due to high levels of capsule expression in these carriage isolates [20]. For recombinant meningococcal vaccines targeting fHbp, rabbit anti fHbp antibodies seem useful tools to anticipate the strain coverage in particular for vaccines containing solely fHbp (LP2086). For 4CMenB/Bexsero, the meningococcal antigen typing system (MATS) approach can be used [25].

Vaccine-induced herd immunity is an essential aspect in controlling meningococcal disease [26]. Our work based on the comparison of carriage isolates and disease isolates suggested that fHbp targeting meningococcal vaccines might reduce, at least in part, the carriage of some hyperinvasive isolates, and thus contribute to establish a herd effect.

## Supporting Information

Table S1 **Characteristics of carriage isolates of *N. meningitidis*.** Phenotypic and genotypic typing data are displayed as well as the levels of expression of fHbp (Z score) and the bactericidal activity of anti-fHbp antibodies ND = non detectable, NA =  non assigned.
(DOC)
