# Supplementary material for: Genetic Diversity and Levels of Expression of Factor H Binding Protein among Carriage Isolates of Neisseria meningitidis
Source: PLoS One. 2014 Sep 23;9(9):e107240. doi: 10.1371/journal.pone.0107240 (PMC4172500; doi:10.1371/journal.pone.0107240)
Supplement: Table S1 — Characteristics of carriage isolates of N. meningitidis. Phenotypic and genotypic typing data are displayed as well as the levels of expression of fHbp (Z score) and the bactericidal activity of anti-fHbp antibodies ND = non detectable, NA = non assigned. (DOC) [file pone.0107240.s001.doc]

**Table S1 : Characteristics of carriage isolates of *N. meningitidis*.**

**Phenotypic and genotypic typing data are displayed as well as the levels of expression of fHbp (Z score) and the bactericidal activity of anti-fHbp antibodies**

**ND=non detectable,** NA= non assigned

| **N° BACTERIO** | **serogroup** | **Genogroup** | **MIC penG** | **serotype** | **Subtype** | ***cnl locus*** | **ST** | **CC** | **fHbp prot** | **fHbp DNA** | **fHbp variant** | **Z score** | **Survival**  **titers** |
| --- | --- | --- | --- | --- | --- | --- | --- | --- | --- | --- | --- | --- | --- |
| 874 | C | C | 0.064 | NT | P1.5,2 | - | 11 | ST-11 complex/ET-37 complex | 19 | 17 | 2 | ND* |  |
| 2995 | B | B | 0.064 | 1 | P1.12,13 | - | 213 | ST-213 complex | 13 | 18 | 3 | ND | 2 |
| 2934 | B | B | 0.094 | 1 | P1.12,13 | - | 213 | ST-213 complex | 13 | 18 | 3 | ND | 2 |
| 2784 | NG | - | 0.064 | NT | P1.7 | + | 53 | ST-53 complex | 9 | 11 | 2 | ND |  |
| 912 | B | B | 0.047 | 1 | P1.14 | - | 7193 | ST-213 complex | 13 | 18 | 3 | ND |  |
| 616 | B | B | 0.125 | NT | P1.4 | - | 5805 | ST-162 complex | 10 | 16 | 2 | ND |  |
| 2818 | Y | B | 0.125 | 1 | P1.6 | - | 7442 | ST-41/44 complex/Lineage 3 | 9 | 11 | 2 | ND |  |
| 1244 | NG | - | 0.047 | NT | P1.6 | + | 2441 | ST-53 complex | 27 | 35 | 1 | ND |  |
| 3148 | NG | - | 0.064 | NT | NST | - | 7445 | NA | 19 | 17 | 2 | ND |  |
| 3009 | NG | - | 0.064 | 4 | P1.7 | + | 53 | ST-53 complex | 9 | 11 | 2 | ND |  |
| 494 | NG | - | 0.094 | NT | P1.7 | + | 53 | ST-53 complex | 9 | 11 | 2 | ND |  |
| 504 | NG | - | 0.094 | NT | P1.7 | + | 53 | ST-53 complex | 9 | 11 | 2 | ND |  |
| 739 | C | C | 0.064 | NT | P1.5,2 | - | 11 | ST-11 complex/ET-37 complex | 19 | 17 | 2 | ND |  |
| 1130 | NG | - | 0.094 | 4 | P1.7 | + | 53 | ST-53 complex | 9 | 11 | 2 | ND |  |
| 1185 | NG | - | 0.094 | NT | NST | + | 1117 | ST-1117 complex | 10 | 16 | 2 | ND |  |
| 1289 | NG | C | 0.064 | 2a | P1.5,2 | - | 11 | ST-11 complex/ET-37 complex | 19 | 17 | 2 | ND |  |
| 1315 | NG | B | 0.380 | 1 | P1.6 | - | 1946 | ST-461 complex | 2 | 20 | 3 | ND |  |
| 1330 | Y | Y | 0.064 | NT | P1.12 | - | 6772 | ST-23 complex/cluster A3 | 8 | 12 | 2 | ND |  |
| 1480 | C | C | 0.047 | NT | P1.5,2 | - | 11 | ST-11 complex/ET-37 complex | 19 | 17 | 2 | ND |  |
| 1937 | C | C | 0.064 | 4 | P1.14 | - | 35 | ST-35 complex | 9 | 11 | 2 | ND |  |
| 1954 | NG | - | 0.047 | NT | P1.7 | + | 53 | ST-53 complex | 9 | 11 | 2 | ND |  |
| 2009 | NG | - | 0.094 | NT | NST | - |  | NA | 8 | 12 | 2 | ND |  |
| 2279 | Y | Y | 0.064 | 15 | P1.16 | - | 6763 | ST-174 complex | 10 | 16 | 2 | ND |  |
| 2352 | B | B | 0.094 | 1 | P1.14 | - | 7429 | ST-213 complex | 13 | 18 | 3 | ND |  |
| 2371 | B | B | 0.064 | 1 | P1.14 | - | 213 | ST-213 complex | 13 | 18 | 3 | ND | 2 |
| 2447 | NG | - | 0.750 | NT | P1.7 | + | 53 | ST-53 complex | 27 | 35 | 2 | ND |  |
| 2688 | Y | Y | 0.032 | NT | P1.12 | - | 6772 | ST-23 complex/cluster A3 | 8 | 12 | 2 | ND |  |
| 2716 | B | B | 0.250 | 4 | P1.14 | - |  | ST-35 complex | 9 | 11 | 2 | ND | 2 |
| 2806 | E | - | 0.047 | NT | P1.5,2 | - | 60 | ST-60 complex | 8 | 12 | 2 | ND |  |
| 2902 | Y | Y | 0.023 | NT | NST | - | 23 | ST-23 complex/cluster A3 | 8 | 12 | 2 | ND |  |
| 3319 | NG | - | 0.190 | 15 | P1.6 | + | 7448 | ST-1136 complex | 7 | 33 | 3 | ND |  |
| 292 | Y | Y | 0.094 | 15 | P1.16 | - |  | ST-174 complex | 10 | 16 | 2 | ND |  |
| 376 | B | B | 0.190 | 1 | P1.6 | - | 1946 | ST-461 complex | 2 | 20 | 3 | ND |  |
| 519 | NG | B | 0.064 | NT | NST | - | 146 | ST-41/44 complex/Lineage 3 | 1 | 24 | 2 | ND |  |
| 576 | B | B | 0.125 | 1 | P1.6 | - | 7188 | ST-41/44 complex/Lineage 3 | 1 | 24 | 2 | ND |  |
| 858 | B | B | 0.380 | 1 | P1.6 | - | 1946 | ST-461 complex | 2 | 20 | 3 | ND |  |
| 1046 | C | C | 0.064 | NT | P1.4,7 | - | 772 | ST-254 complex | 12 | 3 | 1 | ND | 2 |
| 1061 | C | C | 0.064 | 4 | P1.14 | - | 35 | ST-35 complex | 9 | 11 | 2 | ND |  |
| 1115 | NG | Y | 0.047 | NT | NST | - | 23 | ST-23 complex/cluster A3 | 8 | 12 | 2 | ND |  |
| 1126 | NG | B | 0.380 | 14 | P1.7,16 | - | 32 | ST-32 complex/ET-5 complex | 3 | 31 | 1 | ND |  |
| 1201 | Y | Y | 0.047 | NT | NST | - | 23 | ST-23 complex/cluster A3 | 8 | 12 | 2 | ND |  |
| 1226 | C | C | 0.047 | NT | P1.5,2 | - | 11 | ST-11 complex/ET-37 complex | 19 | 17 | 2 | ND |  |
| 1231 | B | B | 0.047 | NT | P1.14 | - | 213 | ST-213 complex | 13 | 18 | 3 | ND | 2 |
| 1311 | NG | C | 0.032 | 2a | P1.5,2 | - | 11 | ST-11 complex/ET-37 complex | 19 | 17 | 2 | ND |  |
| 1610 | B | B | 0.064 | 1 | P1.14 | - |  | ST-213 complex | 13 | 18 | 3 | ND |  |
| 1695 | Y | Y | 0.047 | 15 | P1.5 | - | 1627 | ST-167 complex | 19 | 17 | 2 | ND |  |
| 1770 | B | B | 0.064 | NT | P1.14 | - | 7417 | ST-213 complex | 13 | 18 | 3 | ND |  |
| 2004 | C | C | 0.047 | 4 | P1.14 | - | 35 | ST-35 complex | 9 | 11 | 2 | ND |  |
| 2065 | C | C | 0.032 | NT | P1.5,2 | - | 11 | ST-11 complex/ET-37 complex | 19 | 17 | 2 | ND |  |
| 2121 | Y | Y | 0.047 | 15 | P1.5 | - | 1627 | ST-167 complex | 19 | 17 | 2 | ND |  |
| 2495/CG | C | C | 0.047 | NT | P1.5,2 | - | 11 | ST-11 complex/ET-37 complex | 19 | 17 | 2 | ND |  |
| 2794 | C | C | 0.032 | NT | P1.5,2 | - |  | ST-11 complex/ET-37 complex | 12 | 3 | 1 | ND | 2 |
| 3005 | NG | Y | 0.250 | 15 | P1.9 | - | 1466 | ST-174 complex | 10 | 16 | 2 | ND |  |
| 3008 | Y | Y | 0.250 | NT | P1.4 | - | 1768 | NA | 9 | 43 | 2 | ND |  |
| 3017 | C | C | 0.064 | NT | P1.5,2 | - | 11 | ST-11 complex/ET-37 complex | 19 | 17 | 2 | ND |  |
| 3257 | C | C | 0.047 | NT | P1.5,2 | - | 11 | ST-11 complex/ET-37 complex | 19 | 17 | 2 | ND |  |
| 3293 | Y | Y | 0.047 | 15 | P1.5 | - | 1627 | ST-167 complex | 19 | 17 | 2 | ND |  |
| 3601 | B | B | 0.047 | NT | P1.4,7 | - | 162 | ST-162 complex | 10 | 16 | 2 | -4.42 | 2 |
| 2213 | NG | - | 0.064 | NT | NST | - |  | NA | 8 | 12 | 2 | -2.24 |  |
| 341 | B | B | 0.190 | 1 | NST | - | 6075 | ST-41/44 complex/Lineage 3 | 1 | 24 | 2 | -2.21 | 2 |
| 3542 | Y | Y | 0.094 | 15 | P1.16 | - | 6763 | ST-174 complex | 10 | 16 | 2 | -1.96 |  |
| 2370 | W | W | 0.094 | NT | P1.5,2 | - | 5799 | ST-22 complex | 9 | 11 | 2 | -1.85 |  |
| 2507 | B | B | 0.190 | 1 | P1.1 | - | 7432 | ST-213 complex | 13 | 18 | 3 | -1.80 |  |
| 2673 | E | - | 0.032 | NT | P1.5,2 | - | 7437 | ST-60 complex | 12 | 3 | 1 | -1.79 |  |
| 2630 | NG | - | 0.047 | NT | P1.6 | + | 2441 | ST-53 complex | 27 | 35 | 2 | -1.75 |  |
| 1623 | B | B | 0.125 | NT | P1.6 | - | 414 | ST-41/44 complex/Lineage 3 | 1 | 24 | 2 | -1.74 |  |
| 2409 | E | - | 0.047 | NT | P1.5,2 | - | 60 | ST-60 complex | 12 | 3 | 1 | -1.54 |  |
| 1318 | B | B | 0.250 | 1 | P1.6 | - | 1946 | ST-461 complex | 2 | 20 | 3 | -1.53 |  |
| 1900 | NG | C | 0.047 | NT | P1.5,2 | - | 11 | ST-11 complex/ET-37 complex |  |  |  | -1.37 |  |
| 2715 | W | W | 0.125 | NT | P1.6 | - | 22 | ST-22 complex | 9 | 11 | 2 | -1.33 |  |
| 2407 | E | - | 0.032 | NT | P1.5,2 | - |  | ST-60 complex | 12 | 3 | 1 | -1.12 |  |
| 1609 | B | B | 0.064 | NT | P1.6 | - | 414 | ST-41/44 complex/Lineage 3 | 1 | 24 | 2 | -1.07 |  |
| 2578 | C | C | 0.047 | NT | P1.5,2 | - | 11 | ST-11 complex/ET-37 complex | 19 | 17 | 2 | -1.06 |  |
| 2529 | C | C | 0.047 | 4 | P1.1 | - |  | NA | 1 | 65 | 2 | -0.97 |  |
| 1893 | NG | - | 0.125 | 15 | P1.6 | + | 7418 | ST-1136 complex | 7 | 33 | 3 | -0.97 |  |
| 1658 | NG | C | 0.047 | 1 | NST | - | 212 | ST-212 complex | 1 | 24 | 2 | -0.97 |  |
| 3292 | NG | B | 0.047 | NT | P1.6 | - | 7447 | ST-41/44 complex/Lineage 3 | 1 | 22 | 2 | -0.94 |  |
| 3314 | B | B | 0.125 | NT | P1.6 | - | 414 | ST-41/44 complex/Lineage 3 | 1 | 24 | 2 | -0.94 |  |
| 2765 | NG | C | 0.047 | 1 | NST | - | 7441 | ST-212 complex | 1 | 24 | 2 | -0.91 |  |
| 2598 | NG | B | 0.064 | 1 | P1.6 | - | 7436 | ST-269 complex | 28 | 37 | 2 | -0.83 |  |
| 2484 | Y | Y | 0.380 | NT | P1.4 | - | 1768 | NA | 9 | 43 | 2 | -0.79 |  |
| 2711 | B | B | 0.064 | NT | P1.14 | - | 7439 | ST-213 complex | 47 | 66 | 3 | -0.78 |  |
| 3026 | NG | - | 0.094 | 15 | P1.6 | + | 1136 | ST-1136 complex | 7 | 33 | 3 | -0.77 |  |
| 1607 | E | - | 0.032 | NT | P1.2 | - |  | ST-60 complex | 12 | 3 | 1 | -0.77 |  |
| 1913 | NG | - | 0.125 | 15 | P1.6 | + | 1136 | ST-1136 complex | 7 | 33 | 3 | -0.74 |  |
| 1425 | W | W | 0.250 | NT | P1.6 | - | 184 | ST-22 complex | 9 | 11 | 2 | -0.72 |  |
| 3381 | Y | - | 0.125 | 15 | P1.6 | + | 1136 | ST-1136 complex | 7 | 33 | 3 | -0.69 |  |
| 1287 | NG | - | 0.190 | 15 | P1.6 | + | 1136 | ST-1136 complex | 7 | 33 | 3 | -0.65 |  |
| 2504 | Y | C | 0.064 | 4 | P1.7,13 | - | 7431 | NA | 8 | 12 | 2 | -0.65 |  |
| 597 | NG | B | 0.125 | 1 | P1.6 | - | 7189 | ST-41/44 complex/Lineage 3 | 1 | 24 | 2 | -0.64 |  |
| 1327 | NG | - | 0.047 | 15 | P1.1 | + | 4061 | ST-1136 complex | 7 | 33 | 3 | -0.59 |  |
| 1845 | NG | - | 0.047 | NT | P1.7 | + |  | ST-53 complex | 22 | 9 | 1 | -0.58 |  |
| 1597 | NG | C | 0.047 | 4 | P1.14 | - | 6445 | ST-35 complex | 9 | 11 | 2 | -0.48 |  |
| 3493 | W | W | 0.094 | NT | P1.5,2 | - | 22 | ST-22 complex | 9 | 11 | 2 | -0.44 |  |
| 755 | NG | - | 0,,094 | NT | P1.7 | + | 53 | ST-53 complex | 9 | 11 | 2 | -0.39 |  |
| 3174 | NG | - | 0.125 | 15 | P1.6 | + | 1136 | ST-1136 complex | 7 | 33 | 3 | -0.36 |  |
| 3358 | NG | B | 0.064 | NT | NST | - |  | ST-41/44 complex/Lineage 3 | 1 | 24 | 2 | -0.33 |  |
| 2230 | B | B | 0.250 | 1 | P1.13 | - | 7425 | ST-461 complex | 2 | 20 | 3 | -0.32 |  |
| 2193 | B | B | 0.064 | 15 | P1.7 | - | 7422 | ST-32 complex/ET-5 complex | 10 | 16 | 2 | -0.31 |  |
| 3435 | B | B | 0.047 | 4 | P1.14 | - | 35 | ST-35 complex | 9 | 11 | 2 | -0.28 |  |
| 3521 | NG | Y | 0.047 | NT | NST | - | 23 | ST-23 complex/cluster A3 | 8 | 12 | 2 | -0.28 |  |
| 1603 | NG | - | 0.190 | 15 | P1.12,13 | + | 7186 | ST-1136 complex | 7 | 33 | 3 | -0.26 |  |
| 671 | B | B | 0.380 | 4 | P1.14 | - | 6402 | ST-35 complex | 9 | 11 | 2 | -0.26 |  |
| 2571 | NG | - | 0.125 | NT | P1.7 | + | 7434 | NA** | 7 | 44 | 3 | -0.21 |  |
| 2974 | B | B | 0.047 | 15 | P1.7,16 | - | 32 | ST-32 complex/ET-5 complex | 4 | 7 | 1 | -0.18 |  |
| 2610 | NG | - | 0.032 | NT | NST | + | 1117 | ST-1117 complex | 10 | 16 | 2 | -0.17 |  |
| 2400 | B | B | 0.250 | NT | P1.7,13 | - | 40 | ST-41/44 complex/Lineage 3 | 4 | 45 | 1 | -0.17 |  |
| 2289 | NG | - | 0.125 | 15 | P1.12 | + | 7186 | ST-1136 complex | 7 | 33 | 3 | -0.15 |  |
| 1255 | B | B | 0.250 | 1 | P1.6 | - | 7190 | ST-461 complex | 2 | 20 | 3 | -0.14 |  |
| 2367 | NG | - | 0.032 | 4 | P1.7,13 | - | 103 | ST-103 complex | 8 | 12 | 2 | -0.14 |  |
| 2499 | E | - | 0.032 | NT | P1.2 | - | 60 | ST-60 complex | 12 | 3 | 1 | -0.11 |  |
| 3497 | E | - | 0.047 | NT | P1.5,2 | - | 7452 | ST-60 complex | 12 | 3 | 1 | -0.09 |  |
| 3120 | B | B | 0.250 | NT | NST | - |  | ST-461 complex | 2 | 20 | 3 | -0.09 |  |
| 2262 | B | B | 0.047 | NT | NST | - | 7426 | ST-41/44 complex/Lineage 3 | 1 | 24 | 2 | -0.06 |  |
| 1743 | NG | - | 0.125 | 15 | P1.6 | + | 1136 | ST-1136 complex | 7 | 33 | 3 | -0.05 |  |
| 3510 | W | W | 0.125 | NT | NST | - | 22 | ST-22 complex | 9 | 11 | 2 | -0.03 |  |
| 133 | E | - | 0.032 | NT | P1.5,2 | - |  | ST-60 complex | 12 | 3 | 1 | -0.01 |  |
| 545 | NG | - | 0.064 | NT | P1.5,2 | - | 7185 | ST-334 complex | 12 | 3 | 1 | 0.00 |  |
| 2584 | NG | - | 0.064 | NT | P1.7 | + | 53 | ST-53 complex | 9 | 11 | 2 | 0.01 |  |
| 1932 | Y | Y | 0.250 | NT | P1.4 | - | 1768 | NA | 9 | 43 | 2 | 0.02 |  |
| 1390 | NG | - | 0.125 | 15 | P1.6 | + | 1136 | ST-1136 complex | 7 | 33 | 3 | 0.02 |  |
| 601 | NG | - | 0.047 | 1 | P1.6 | - | 963 | NA | 12 | 3 | 1 | 0.03 |  |
| 2455 | X | - | 0.190 | 15 | P1.6 | + | 1136 | ST-1136 complex | 7 | 33 | 3 | 0.05 |  |
| 1358 | NG | - | 0.190 | 15 | P1.14 | + | 7192 | ST-1136 complex | 7 | 33 | 3 | 0.07 |  |
| 2521 | B | B | 0.250 | 1 | P1.6 | - | 7433 | ST-461 complex | 2 | 20 | 3 | 0.11 |  |
| 1769 | NG | - | 0.190 | 15 | P1.6 | + | 1136 | ST-1136 complex | 7 | 33 | 3 | 0.11 |  |
| 1799 | W | W | 0.190 | NT | P1.6 | - | 184 | ST-22 complex | 9 | 11 | 2 | 0.11 |  |
| 2799 | NG | - | 0.190 | 4 | P1.5,2 | - |  | ST-254 complex | 12 | 3 | 1 | 0.12 |  |
| 955 | NG | - | 0.250 | 15 | P1.6 | + | 1136 | ST-1136 complex | 7 | 33 | 3 | 0.14 |  |
| 2698 | NG | - | 0.064 | 15 | P1.6 | + | 1136 | ST-1136 complex | 7 | 33 | 3 | 0.15 |  |
| 2117 | C | - | 0.125 | 15 | P1.6 | + | 1136 | ST-1136 complex | 7 | 33 | 3 | 0.19 |  |
| 981 | NG | - | 0.032 | 15 | P1.6 | + | 198 | ST-198 complex | 26 | 34 | 3 | 0.21 |  |
| 2694 | X | X | 0.190 | NT | P1.5,2 | - | 7438 | NA | 15 | 4 | 1 | 0.21 |  |
| 2593 | NG | - | 0.064 | 1 | P1.6 | + | 7435 | ST-41/44 complex/Lineage 3 | 1 | 24 | 2 | 0.23 |  |
| 2625 | NG | - | 0.047 | NT | P1.5,2 | - | 60 | ST-60 complex | 12 | 3 | 1 | 0.24 |  |
| 2662 | B | B | 0.047 | NT | P1.9 | - | 3752 | ST-41/44 complex/Lineage 3 | 1 | 24 | 2 | 0.24 |  |
| 2073 | B | B | 0.125 | 4 | P1.14 | - |  | ST-162 complex | 19 | 17 | 2 | 0.30 |  |
| 2211 | B | B | 0.064 | NT | NST | - | 7424 | ST-41/44 complex/Lineage 3 | 1 | 24 | 2 | 0.31 |  |
| 1590 | NG | - | 0.064 | 4 | P1.12 | + | 3275 | NA | 22 | 9 | 1 | 0.32 |  |
| 3000 | NG | C | 0.047 | 1 | NST | - |  | ST-212 complex | 1 | 24 | 2 | 0.32 |  |
| 3616 | B | B | 0.047 | 15 | P1.7 | - | 32 | ST-32 complex/ET-5 complex | 4 | 7 | 1 | 0.32 |  |
| 1562 | NG | C | 0.047 | 4 | P1.7,14 | - | 35 | ST-35 complex | 9 | 11 | 2 | 0.36 |  |
| 2180 | NG | - | 0.064 | NT | NST | - |  | NA | 8 | 12 | 2 | 0.36 |  |
| 744 | NG | - | 0.125 | 15 | P1.6 | + | 1136 | ST-1136 complex | 7 | 33 | 3 | 0.41 |  |
| 124 | NG | - | 0.190 | 15 | P1.6 | - | 1136 | ST-1136 complex | 7 | 33 | 3 | 0.42 |  |
| 2917 | NG | X | 0.064 | NT | P1.5,2 | - | 7444 | NA | 15 | 4 | 1 | 0.44 |  |
| 1569 | NG | X | 0.047 | NT | P1.16 | - |  | ST-1157 complex | 12 | 3 | 1 | 0.46 |  |
| 2326 | NG | - | 0.125 | NT | P1.7 | + | 53 | ST-53 complex | 9 | 11 | 2 | 0.47 |  |
| 647 | NG | - | 0.094 | 15 | P1.6 | + | 1136 | ST-1136 complex | 7 | 33 | 3 | 0.51 |  |
| 533 | NG | - | 0.094 | NT | P1.16 | - | 5063 | NA | 15 | 4 | 1 | 0.52 |  |
| 1267 | NG | X | 0.064 | NT | P1.16 | - | 1157 | ST-1157 complex | 12 | 3 | 1 | 0.52 |  |
| 2413/CG | NG | - | 0.064 | NT | P1.5,2 | - | 7430 | ST-60 complex | 12 | 3 | 1 | 0.53 |  |
| 507 | NG | X | 0.094 | NT | NST | - | 5421 | NA | 15 | 4 | 1 | 0.54 |  |
| 852 | NG | - | 0.190 | 15 | P1.6 | + | 1136 | ST-1136 complex | 7 | 33 | 3 | 0.55 |  |
| 1632 | B | B | 0.032 | 15 | P1.7,16 | - | 32 | ST-32 complex/ET-5 complex | 4 | 7 | 1 | 0.56 |  |
| 117 | NG | W | 0.190 | NT | P1.6 | - | 184 | ST-22 complex | 9 | 11 | 2 | 0.61 |  |
| 1876 | NG | C | 0.064 | 4 | P1.14 | - | 35 | ST-35 complex | 9 | 11 | 2 | 0.61 |  |
| 280 | B | B | 0.064 | NT | P1.9 | - | 3752 | ST-41/44 complex/Lineage 3 | 1 | 24 | 2 | 0.64 |  |
| 878 | NG | B | 0.125 | 1 | P1.6 | - | 414 | ST-41/44 complex/Lineage 3 | 1 | 24 | 2 | 0.64 |  |
| 128 | NG | B | 0.064 | 1 | P1.6 | - | 414 | ST-41/44 complex/Lineage 3 | 1 | 24 | 2 | 0.66 |  |
| 492 | B | B | 0.064 | 15 | P1.7 | - | 32 | ST-32 complex/ET-5 complex | 4 | 7 | 1 | 0.66 |  |
| 913 | NG | - | 0.250 | NT | P1.5 | - |  | ST-60 complex | 12 | 3 | 1 | 0.68 |  |
| 2076 | NG | B | 0.064 | 1 | P1.6 | - | 492 | ST-269 complex | 28 | 37 | 2 | 0.73 |  |
| 1820 | B | B | 0.047 | 4 | P1.15 | - | 944 | ST-41/44 complex/Lineage 3 | 1 | 24 | 2 | 0.73 |  |
| 3409 | NG | B | 0.047 | 15 | P1.7,16 | - | 7451 | ST-32 complex/ET-5 complex | 4 | 7 | 1 | 0.79 |  |
| 2449 | X | B | 0.064 | NT | NST | - | 437 | ST-41/44 complex/Lineage 3 | 5 | 1 | 1 | 0.80 |  |
| 1515 | B | B | 0.032 | NT | P1.7,2 | - | 7191 | NA | 1 | 24 | 2 | 0.83 |  |
| 2202 | NG | B | 0.047 | 15 | P1.7,16 | - | 7614 | ST-32 complex/ET-5 complex | 4 | 7 | 1 | 0.83 |  |
| 119 | B | B | 0.047 | 15 | P1.7,16 | - |  | ST-32 complex/ET-5 complex | 4 | 7 | 1 | 0.94 |  |
| 368 | NG | - | 0.064 | NT | P1.16 | - | 5063 | NA | 15 | 4 | 1 | 0.97 |  |
| 589 | NG | - | 0.125 | 4 | P1.12 | + | 3275 | NA | 22 | 9 | 1 | 0.99 |  |
| 877 | NG | B | 0.094 | 1 | P1.6 | - | 414 | ST-41/44 complex/Lineage 3 | 1 | 24 | 2 | 1.04 |  |
| 3387 | X | - | 0.125 | 15 | P1.6 | + | 2236 | ST-198 complex | 5 | 36 | 1 | 1.07 |  |
| 1617 | NG | B | 0.064 | 14 | P1.7,16 | - | 32 | ST-32 complex/ET-5 complex | 7 | 14 | 3 | 1.17 |  |
| 3386 | NG | - | 0.125 | 15 | P1.6 | + | 7450 | ST-1136 complex | 5 | 36 | 1 | 1.18 |  |
| 1399 | NG | - | 0.125 | 15 | P1.6 | + | 823 | ST-198 complex | 5 | 36 | 1 | 1.23 |  |
| 3266 | NG | - | 0.047 | 15 | P1.6 | + | 7446 | ST-198 complex | 5 | 36 | 1 | 1.30 |  |
| 2850 | NG | B | 0.190 | 4 | P1.5,2 | - | 7443 | ST-32 complex/ET-5 complex | 3 | 26 | 1 | 1.47 |  |
| 2505 | NG | B | 0.064 | NT | NST | - | 269 | ST-269 complex | 11 | 2 | 1 | 1.50 |  |
| 537 | NG | - | 0.250 | 4 | P1.6 | - | 7194 | ST-32 complex/ET-5 complex | 3 | 31 | 1 | 1.71 |  |
| 2866 | B | B | 0.064 | 14 | P1.7,16 | - |  | ST-32 complex/ET-5 complex | 3 | 31 | 1 | 1.71 |  |
| 3512 | B | B | 0.094 | 14 | P1.7,16 | - | 32 | ST-32 complex/ET-5 complex | 3 | 31 | 1 | 1.72 |  |
| 3503 | B | B | 0.064 | 14 | P1.7,16 | - | 6807 | ST-32 complex/ET-5 complex | 3 | 31 | 1 | 1.78 |  |
| 2963 | NG | B | 0.190 | 14 | P1.7,16 | - | 32 | ST-32 complex/ET-5 complex | 3 | 31 | 1 | 1.85 |  |
| 3180 | B | B | 0.047 | 14 | P1.7,16 | - | 32 | ST-32 complex/ET-5 complex | 3 | 31 | 1 | 1.87 | 16 |
| 2943 | B | B | 0.032 | 14 | P1.7,16 | - | 32 | ST-32 complex/ET-5 complex | 3 | 31 | 1 | 1.87 |  |
| 2777 | NG | - | 0.032 | 4 | NST | - |  | ST-254 complex | 37 | 67 | 1 | 1.89 |  |
| 2574 | NG | B | 0.380 | 4 | P1.15 | - | 33 | ST-32 complex/ET-5 complex | 3 | 31 | 1 | 2.07 |  |
